# Somnambulism

**Published:** 1887-02

**Authors:** 


HALL'S

### JOURNAL OF HEALTH

TRUTH DEMANDS NO SACRIFICE; ERROR CAN MAKE NONE.

VOL. 34. FEBRUARY, 1887. No 2.

### SOMNAMBULISM.

"Powers there are
That touch each other to the quick, in modes
Which the gross world no sense hath to perceive."

The term somnambulism is compounded of two Latin words, which signify sleep-walking, but in common acceptation it has acquired a wider meaning, and comprehends the various phenomena which occur in connection with sleep-walking. Hence somnambulism may be demonstrated a *sleeping trance*, accompanied by physical exertions or movements intelligently guided, usually superinduced by extreme mental activity in the direction of the manifestation. In these states of unconscious mental and physical activity, all the powers of the mind seem to be concentrated upon a particular object, or as it is held by a majority of writers upon this subject, certain mental faculties are more keenly alive than at any other period, whilst others remain, for the time, in a state of suspension.

Thus is it that inventors, musicians, authors and mathematicians have, in such a state, been able to achieve successes quite out of the reach of their normal perceptions.

In the form now recognized by magnetizers, somnambulism was first observed by the Marquis de Puysègur, near Sissons, in 1784, and Dr. Bretrand, a member of the Faculty of Medicine of Paris, in treating of the subject says: "The somnambule acquires new perceptions furnished by interior organs, and the succession of these perceptions constitutes a new life, differing from that which we habitually enjoy; in that new life come to light phases of knowledge differing from those which our ordinary sensations convey to us."

So, too, Robert Dale Owen in his "Debatable Land," says of himself: "I have, on many occasions, verified this phenomenon of what may be called double consciousness, attended by exaltation of intelligence in the abnormal state. But," continues this author, "the analogy between these and the various phases of intellectual and physical exaltation, religious ecstacy, involuntary hypnotism, spontaneous trance, is so close that one cannot reasonably deny the connection of one with the other."

Dr. S. B. Brittan, whose examination into the facts and philosophy of psychic phenomena, was as far-reaching as any modern investigator, agrees with Mr. Owen, as the following passage, quoted from his "Man and His Relations" demonstrates. "The vision of the somnambulist and the clarivoyance developed in a state of magnetic coma, are essentially the same and may be equally clear and reliable. The somnambulist generally walks with his eyes wide open, though this is not always the case ; but whether the lids be opened or closed, the pupil is invariably dilated to its utmost capacity. The six muscles that move the eye appear to be motionless, and the expression is fixed, vacant and glassy. In this state the eye is evidently useless as the organic instrument of vision, since the optic nerve no longer conveys images of external objects to the mind. The pupil, though exposed to the solar rays, will never contract in the smallest appreciable degree, nor is the influence of the strongest light perceptible in the action of the glandulæ lachrymoles."

"The eyes are open,
"But their sense is shut."

Walking in sleep, however, is not the most marvelous example of this phenomenon, for the somnambulist not only walks, but rides, climbs, and with eyes shut or insensible, makes his way, with apparent indifference to danger, into positions perilous to one in the full exercise of his ordinary senses. Strange as it may appear, it is well authenticated that persons in this state have performed long journeys on foot and on horseback, keeping to the highway, paying tolls and avoiding obstsuctions. They have, by night, descended into coal mines, ascended to the roofs of houses, and climbed rocky cliffs in pursuit of some ruling object. Artizens too, have pursued their various avocations without the aid of artificial light, beginning at the intermitted point of their unfinished labors, and doing their work with the ability and skill of their waking hours, but retaining no recollection of it afterwards.

There is an account of a cook who, having prepared himself a salad, simple cabbage was substituted for it, which he ate with equal relish. This would seem to imply that the sense of taste was meanwhile dormant.

Dr. George Moore, member of the College of Physicians, London, in a work entitled "The Power of the Soul over the Body," 1835, relates a number of curious somnambulic occurrences. "A man," writes he, "has been known to fall asleep while walking at the end of a fatiguing journey, and he could not be roused from his sleep without great difficulty, although he continued to walk in company with his friends for a considerable distance." It is also a well established fact, that in the disastrous retreat of Sir John Moore toward the Spanish coast, before the battle of Corunna, many of the soldiers fell asleep, yet continued to march with their comrades. This circumstance has been so often avouched by writers, that it may be said to be firmly established as a physiological fact. "In these cases," says Dr. Moore, "we observe that the mind controls the actions of the voluntary muscles, and continues attending to visible objects, without employing the sense of the sight, and apparently receives impressions of sound, while the auditory nervous apparatus is quite insensible. It may be true that certain portions of the brain sleep while other portions remain awake; but what does that signify? Can one part of the brain subserve the purpose of the other parts, and those organs which phrenologists appropriate to thought, furnish a substitute in their own action for that of the instruments of vision and of hearing?" * * * "It is evident that the integrity of mental action is not dependent on the waking activity of the brain, or at least of that portion of it which is more immediately connected with the senses; for we possess incontrovertible evidence that the mind is sometimes employed more clearly in profound sleep than when the attention is in any degree directed to the senses."

Dr. Brittan, who in his volume of a later date fully indorses the views of Dr. Moore upon these interesting subjects, has presented a number of interesting cases as coming within his personal observation. He says. "Mrs. Newton, a relative of the writer, was a skillful seamstress, and was accustomed to the unconscious use of her needle for hours at night, when there was no light in her room. A friend, who was an accomplished horseman, often rode many miles while he was in a profound slumber." Again he says: "Some years since, while a young lady—a member of the author's family—was at school, it was observed that she succeeded in her Latin exercise, without, apparently, devoting much time or attention to the subject. At length the secret of her easy progress was discovered. She was observed to leave her room at night, and—taking her class books—she proceeded to a certain place on the bank of a small stream, where she remained but a short time, and then returned to the house. In the morning she was invariably unconscious of what had occurred during the night, but a glance at the lesson for the day usually resulted in the discovery that it was already quite as familiar to her mind as household words." Similar facts are published in "Notes and Queries" of a young student from the Greek Archipelego, who, though extremely stupid, displayed great proficiency in his Latin and geometrical exercises, which were found to be acquired during the somnambulic state. A case is reported by Professor Soave, of an apothecary's clerk, who not only walked while asleep, but would light his fire, pursue his studies, examining authorities, classify botanical specimens, engage in animated controversies with his employer or Prof. Soave, on chemistry and other scientific themes, and indeed, perform any duty or service that he was accustomed to do in his waking hours. He would also carefully compound medicines, according to the prescriptions that were before him, but conscientiously declined filling false prescriptions, or such as would be likely to injure the patient.

Dr. Gall, the eminent psychologist, gives an account of a miller who was in the habit of rising every night and running his mill in the usual business way. There is also an account by Mertinet of a saddler who was accustomed to work at his trade while sleeping.

The following case is related in the first volume of "The Lancet": George Davies, aged sixteen years, in the service of Mr. Hewson, butcher, of Bridge Road, Lambeth, being fatigued, bent forward in his chair, and, resting his forehead on his hands, soon fell asleep. After ten minutes he started up, went for his whip, put on his spurs, and went to the stable. Not finding his own saddle in the usual place, he returned to the house and asked for it. Being asked what he wanted with it, he replied, "to go his rounds." He returned to the stable, got on his horse without his saddle, and was proceeding to the street, when, with much difficulty and force, he was removed from his horse. He fancied himself stopped at the turnpike gate, took sixpeuce out of his pocket to be changed, and, holding out his hand for the change, the sixpence was returned to him. He immediately observed, "None of your nonsense; that is the sixpence again—give me my change." When two-and-a-half pence was given to him, he counted it and said: "None of your nonsense; that is not right. I want a penny more," which was the proper change. He then said, "give me my *castor*," and began to whip and spur and get on his way.

Dr. Abercrombie relates that an eminent lawyer, after several days of intense attention to a subject of great importance which had been submitted to him, got up in the night during his sleep and wrote a lengthy paper. The following morning he said he had had a most interesting dream, and would give anything to regain the train of thought which had occupied his mind. Upon going to his writing desk he found his opinion clearly and luminously written out.

A former Archbishop of Bordeaux has given an account of a student who composed a sermon and wrote music in the somnambulic state; reading over his work, scratching out lines, substituting others, and interlining omitted words, with the care and precision of a practised author, and this he persevered in doing, although a sheet of pasteboard was interposed between the eyes of the sleeper and his paper, thus proving conclusively that he was copying mental images, unaided by his ordinary perceptions.

The foregoing examples have been selected almost at random, from a mass of similar ones, which have been preserved to us.

The subject is a very interesting one, as presenting many phases of mental phenomena, wholly distinct from the conscious exercise of the intellectual faculties and the subordination thereto of the physical organism in a normal state. It will be further treated in our next issue under the head of somniloquism, or talking in one's sleep, which is only another phase of the phenomenon presenting evidences of mental exertion by system and method, at times when all the ordinary avenues to the interior perceptions are barred, and the physical man is withdrawn from the theatre of his activity, wherein he plays his customary round.

